# What Do Men Want from a Health Screening Mobile App? A Qualitative Study

**DOI:** 10.1371/journal.pone.0169435

**Published:** 2017-01-06

**Authors:** Chin Hai Teo, Chirk Jenn Ng, Alan White

**Affiliations:** 1 Department of Primary Care Medicine, Faculty of Medicine, University of Malaya, Kuala Lumpur, Malaysia; 2 Centre for Men’s Health, Leeds Beckett University, Leeds, United Kingdom; University of Texas at San Antonio, UNITED STATES

## Abstract

There is a lack of mobile app which aims to improve health screening uptake developed for men. As part of the study to develop an effective mobile app to increase health screening uptake in men, we conducted a needs assessment to find out what do men want from a health screening mobile app. In-depth interviews and focus group discussions were conducted with 31 men from a banking institution in Kuala Lumpur. The participants were purposely sampled according to their job position, age, ethnicity and screening status. The recruitment was stopped once data saturation was achieved. The audio-recorded interviews were transcribed verbatim and analyzed using thematic approach. Three themes emerged from the analysis and they were: content, feature and dissemination. In terms of the content, men wanted the app to provide information regarding health screening and functions that can assess their health; which must be personalized to them and are trustable. The app must have user-friendly features in terms of information delivery, ease of use, attention allocation and social connectivity. For dissemination, men proposed that advertisements, recommendations by health professionals, providing incentive and integrating the app as into existing systems may help to increase the dissemination of the app. This study identified important factors that need to be considered when developing a mobile app to improve health screening uptake. Future studies on mobile app development should elicit users’ preference and need in terms of its content, features and dissemination strategies to improve the acceptability and the chance of successful implementation.

## Introduction

Health screening is a key component in disease prevention framework. Through regular health screening, one can detect diseases and identify risk factors early when there is still a window of opportunity for interventions before the disease worsens. Despite the importance of health screening, the uptake remains low, particularly in men [1–4], who face barriers related to individual, social, health system, healthcare professional and screening procedural factors. At the individual level, lack of knowledge, lack of symptom, fear of positive results, masculinity attributes and lack of time are common barriers to health screening in men [5, 6]. Social stigma and negative peer influence also hindered men from going for screening [7, 8]. Other factors such as poor accessibility to screening services, cost, lack of physician’s recommendation and uncomfortable screening procedure also contribute to the low uptake of health screening in men [5, 6, 9]. There is a strong imperative to get men engaged in screening as they have been found to be particularly susceptible to ill-health and premature death as a result of conditions that are readily identifiable and treatable if picked up soon enough [10–14].

Many interventions have been developed to increase the uptake of health screening in men. They are often delivered through health education workshops, partner’s involvement, printed messages, reminder call and videos [15–20]. However, these interventions are costly, labor intensive and the dissemination may be limited. Increasingly, information communication technology (ICT)-based interventions, such as web-based decision aids and social media, are being used to improve screening uptake, particularly in hard-to-reach men [21, 22]. To date, few studies have reported on the development and effectiveness of using mobile app to promote health screening.

Health-related mobile apps are increasingly being used and mobile health (mHealth) has become an important tool to improve healthcare. mHealth is able to remove geographical and temporal barriers; it helps to deliver just-in-time healthcare to people at their preferred location [23]. Men, especially the younger group, tend to spend considerable amount of time on their mobile phone. In 2015, on average, both Americans and Malaysians spent about three hours on their mobile devices every day [24, 25]. This creates an opportunity for mobile apps to be used as a platform to potentially improve knowledge and increase uptake of health screening in men.

Several studies have reported that mobile apps are effective in modifying health behavior and improving health status. For example, mobile apps have been found to promote healthy diet and physical activity; improve coping with depression; reduce self-injurious thoughts and behaviors; and reduce medication error [26–29]. Nevertheless, among 165,000 health apps that are available to consumers in 2015, only 12% account for 90% of consumer downloads [30]. Moreover, users have reported that they stopped using some mobile health apps because they had high data entry burden, not interesting, too confusing and did not meet users’ needs [31]. Therefore, it is important to identify users’ preference and needs before developing a mobile app to improve its acceptability and effectiveness [32, 33].

Recent reviews on mobile health apps did not find any health screening-related app. Most of the health apps were developed for healthcare professional rather than for public or patients [34, 35]. A search in app stores found that most of the health screening mobile apps are disease-specific; few provide a ‘one-stop platform’ for comprehensive health screening. For example, the Electronic Preventive Services Selector (ePSS) app is a comprehensive screening mobile app which was developed based on the USPSTF’s recommendations [36]. However, this app targets clinicians and the content is not written for lay people. As part of the study to develop an effective mobile app to increase men’s health screening uptake, we conducted a needs assessment and interviewed men to find out what do they want from a health screening mobile app.

## Materials and Methods

### Study Design and Context

This qualitative study used the interpretive descriptive approach to explore what men want in a health screening mobile app. We conducted semi-structured in-depth interviews (IDIs) and focus group discussions (FGDs) with young men in Kuala Lumpur (KL), Malaysia. KL is the capital of Malaysia with good healthcare accessibility. It is a fast-paced city with a highly competitive working environment. Since, the app is mainly intended for the hard-to-reach men (who are less likely to seek healthcare) in the community, we chose healthy working men, specifically men who are working in a banking institution in view of the stressful and sedentary nature of their job. This study was approved by the University of Malaya Medical Centre Medical Ethics Committee (MECID NO: 201410–701).

### Sampling and Recruitment

We used purposive sampling to recruit men from different age, ethnicity, job position and screening status in order to achieve maximal variation. The participant must also have a smart phone. We contacted a banking institution and sought approval to conduct this study with the staff. The human resource department helped to send emails to all male staff in the organization to invite them to participate in this study. We then made appointments with the participants and conducted the IDIs and FGDs at their workplace. For FGDs, we delimited the group by job position to ensure homogeneity, so that the participants were comfortable discussing and disclosing their views without hierarchical influences.

### Data Collection

Two researchers who were trained in qualitative interviewing and have multilingual ability conducted the IDIs and FGDs. The FGDs trigger interactions and take advantage of group dynamics while the IDI allows the researchers to explore more personal or sensitive issues in depth. The findings from both methods can also be used as a form of triangulation. The IDIs and FGDs were conducted in the language familiar to the group or participant. One of the researchers took field notes while the other led the interviews. To initiate an interview, the participants were first asked whether they were using any health-related mobile app (including health screening) and if so, to describe their experience using the app. We probed for any pros and cons of the app; what characteristics they did and didn’t like regarding the app; what made them keep using or deleted the app. Then, we explained our intention to develop an app to promote health screening. We asked their opinions about the idea and their suggestions of what to be included in the app. Lastly, we asked the participants how to spread and make men download and use the app. Written consent was obtained from all participants and the interviews were audio-recorded. The recruitments and interviews were conducted until data saturation was achieved.

### Data Analysis

All the recorded interviews were transcribed verbatim and the NVivo 10 software was used to manage the data. All names of the participants were coded in the transcripts to ensure anonymity. We analyzed the data using the thematic approach. First of all, two researchers read and reread the first transcript (IDI), second transcript (FGD) and field notes to familiarize themselves with the data. Then, the researchers independently performed open coding, where codes were assigned to each phrase, sentence or paragraph of the transcripts based on the study objectives. Subsequently, axial coding was performed, where the existing codes were combined to form bigger themes according to the relationship found between and within the codes. All researchers met to compare the similarities and differences in the analysis. Any differences were resolved through consensus and this was confirmed by the third researcher. One researcher then continued to code the remaining transcripts and discussed any newly emerged codes with the research team. The researchers also performed constant comparison throughout the analysis to form the final framework. The researchers constantly reflected on their background and roles throughout all phases of the study to avoid potential biases in the results.

## Results

**Fig 1:**
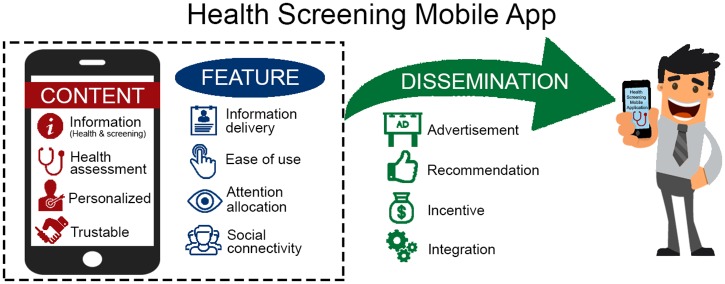
Three main themes of what men want from a health screening mobile app.

Eight IDIs and five FGDs involving 31 men were conducted from July to November 2015. The summary of participants characteristics are shown in Table 1 and the detailed characteristics with participant code are presented in Table 2. Three themes emerged from the analysis and they were: Content, Feature and Dissemination. There were four sub-themes under each of the theme as illustrated in Fig 1.

**Table 1:** Characteristics of all participants.

Characteristic	Number	Percentage (%)
**Age**		
20–29	11	35.5
30–39	10	32.3
40–49	5	16.1
50–59	4	12.9
60–69	1	3.2
**Ethnicity**		
Malay	14	45.2
Chinese	12	38.7
Indian	3	9.7
Others	2	6.5
**Job Position**		
Senior Manager	8	27.8
Officer	7	22.6
Sales Advisor	9	29.0
Clerk	7	22.6
**Education level**		
Primary school	1	3.2
Secondary school	4	12.9
Certificate/Diploma	8	25.8
Degree	17	54.8
Postgraduate	1	3.2
**Regular screening**		
Yes	13	41.9

**Table 2:** Participant code and characteristic.

Participant code*	Age group	Ethnicity	Position	Education	Regular screening
I1	30–39	Indian	Officer	Degree	No
I2	30–39	Other	Senior manager	Postgraduate	No
I3	50–59	Indian	Manager	Certificate/Diploma	Yes
I4	40–49	Malay	Clerk	Primary school	No
I5	20–29	Malay	Sales advisor	Degree	No
I6	30–39	Chinese	Senior manager	Degree	Yes
F1	50–59	Chinese	Senior manager	Degree	Yes
F2	40–49	Malay	Senior manager	Degree	No
I7	60–69	Chinese	Senior manager	Certificate/Diploma	Yes
F3	50–59	Malay	Clerk	Secondary school	No
F4	40–49	Malay	Clerk	Secondary school	No
F5	40–49	Malay	Clerk	Secondary school	No
F6	30–39	Malay	Clerk	Secondary school	No
F7	30–39	Indian	Clerk	Certificate/Diploma	Yes
F8	30–39	Chinese	Officer	Degree	No
F9	20–29	Malay	Officer	Degree	No
F10	20–29	Malay	Officer	Certificate/Diploma	No
F11	20–29	Malay	Officer	Degree	No
F12	20–29	Malay	Clerk	Certificate/Diploma	No
F13	20–29	Malay	Officer	Degree	No
I8	40–49	Malay	Officer	Degree	Yes
F14	50–59	Malay	Senior manager	Degree	Yes
F15	30–39	Other	Senior manager	Degree	No
F16	30–39	Chinese	Sales advisor	Degree	Yes
F17	20–29	Chinese	Sales advisor	Certificate/Diploma	Yes
F18	20–29	Chinese	Sales advisor	Certificate/Diploma	Yes
F19	20–29	Chinese	Sales advisor	Degree	Yes
F20	30–39	Chinese	Sales advisor	Degree	Yes
F21	30–39	Chinese	Sales advisor	Certificate/Diploma	Yes
F22	20–29	Chinese	Sales advisor	Degree	No
F23	20–29	Chinese	Sales advisor	Degree	No

* Note: I = IDI; F = FGD

### Theme 1: Content

#### Information

The participants did not know much about health screening and the doctors often did not have time to explain to them due to short consultation time. They suggested that the screening health app should include information about their health risks, benefits and risks of health screening as well as screening services available to them. Besides screening, the app should provide additional health information such as advice on fitness and healthy diet.

“You have to put in what is health and explain it. People know that healthy is no pain or symptom. Most people don’t know much about screening.”

(F3, translated from Malay)

“If you don’t provide the explanation, one will be like, ‘it [screening] can wait’. I do not know what is the impact and risk.”

(F3, translated from Malay)

“Some kind of advice or comments saying that if men doing regular check-up, or regular exercise, you would reduce what kind of disease, and by how many percent.”

(I6)

“Some kind of a suggestion to select where to go, which one we prefer to go, which one is nearer to our home? Is it a trustworthy doctor or not? Reliable or not?”

(I7)

“I mean you can also instead of just encouraging people to come for health screening, you can also have some other things like talking to people about fitness level, things they can do to keep themselves healthy, you know. So when they look at it, they will also look at some of the health screening that need to be done. So you need to link up food and also fitness. Because I think all these three work hand in hand.”

(I3)

#### Health assessment

The participants in this study felt that an ideal health screening app should have functions that can directly measure and assess their health status. The app should provide the convenience of doing the screening at home in privacy rather than going to the doctor. Most of the participants proposed that the screening app should be able to perform all relevant screening tests, for example heart rate measurement. In addition, this must include assessment of mental and sexual health, which are often not screened by the doctors. Besides for screening purpose, the participants proposed that the app should also include diagnostic function.

“I would love to be able to have that access to do tests on my own, from perhaps at home, like let's say there's a function that, take a deep breath and hold it and then check. What is the heart rate, you know, and then you key in. What's the color of your pee, is it red? or is it yellow or white? And then there should be a button there, ‘Diagnosed’. Then the app will feedback and say ‘Ok, you are having this, this and this.’ Probably can give me an immediate advice that, 'You probably just did not drink a lot of water, you need to drink water' or probably, 'This is a very complicated disease, you need to go and check with the doctor who could advise you further and suggested hospital… doctors…', you know. That would be very useful. I don't mind paying for an app. Fifty Ringgit (USD 13) for that.”

(I2)

“I think I want to check for mental health, stress, depression. These may have an impact when you want to drive, or operate machines. Like sexual health too, sometimes we have problems, not strong. I think this is also important for men. Assess and suggests ways to be stronger or ways to prevent erectile dysfunction. Men like this [kind of assessment], can attract attention.”

(F4)

#### Personalized

The information provided by the app must be tailored. The participants did not want to be overloaded with information but preferred the app to provide individualized feedback and advice on their health. For example, the app should be able to provide information on the user’s health risks, recommend which screening tests the user should undergo, where is the nearest screen center and what actions the user needs to take to stay healthy. The app must be gender-sensitive and is developed specifically for men.

“It will give information, but not full of information. We have to input our health profile, then it will feedback to us. You have to go for this screening and that screening. People who use this app will know, I need to do this, this and this.”

(F4)

“If want to screen, screen for what, when, how frequent. Like me, I obviously don’t know. When do I need to go for screening when I am healthy. What I know now, if healthy, no need, if sick, we go.”

(F6, translated from Malay)

“What's the point of telling this person that you have this and this problem but don't give them a solution after that. You have to also give them solutions so that they can use one app for both.”

(I3)

“To me, I prefer a male-specific app, because it means that, there must have been some thoughts going into it. To the fact that this is only for men's illness.

(F1)

#### Trustable

The participants preferred a health screening app that they can trust. It must be able to keep their personal information especially medical data in a secured manner. In addition, the app must contain up-to-date information and come from a credible source such as the government or professional bodies.

“Ok but I will only follow [the advice] if I feel that the source is credible you know, those that have scientific basis.”

(F1)

“If like approved by the government or professional bodies, maybe people will download it more.”

(F13)

### Theme 2: Feature

#### Information delivery

The participants suggested that the app should provide succinct information and use laymen terms. They also found pictures or video easier to understand compared to text. Some emphasized that health messages must be delivered in a sensitive manner so that it does not cause emotional harm to the users. Language was another important issue raised by the participants. There should be an option to select the language they preferred.

“It cannot be too lengthy you know. You might not have the chance and time to read all the detailed information. Concise and simplified, otherwise, let’s say you give me 10 selections, I will be cracking my head, which one should I go? Maybe you reduce it to 4 or 5, then I can make a faster decision on that.”

(I7)

“For the explanation, it may be good if there is a video or pictures.”

(F3)

“Some people are not good in English, often misinterpreted after translating to Malay, especially the elderly, they don’t understand. We have Malay, Chinese and Indian in Malaysia, better make it in two versions, English and Malay.”

(F4)

“I think you, you need to start off by saying your benefits first before you even get information from them you know, like ‘this app will be able to do this and how it can help you, then in order for us to gauge your health, these are the basic information we need from you.’ You don't straight away shoot them with questions as that will demoralize them. You got to use a nicer approach and make sure that your words and all are pleasant. Don't hit a person too hard like telling them ‘I think you have diabetes’ as that might affect a person emotionally and he or she may never use that app again.”

(I3)

#### Ease of use

Apps that imposed a taxing data entry process are undesirable. The participants suggested that the app should be able to detect health information automatically from devices such as wearables, online account or a hospital database.

“Keying in the data is a hassle for me. I mean unless it can detect automatically. Something that connects to data. It's like connecting GPS data you know; you store somewhere that I don't have to do anything about it. Then fine.”

(F1)

“I think for an app, if I need to type so much information, it won’t be so convenient. People are most concerned about data entry burden when using app nowadays. People want something fast. Like wristband for sport, it can detect your heart rate, maybe auto-extract data from these things.

(F23, translated from Mandarin)

#### Attention allocation

The participants suggested several ways to ensure that the app being kept and used by the users. Reminder was the most common method mentioned by them. The app should not only be able to remind the user on upcoming health screening date, it should provide daily or weekly reminder on ways to improve health.

“One more thing is if the app can provide reminder for us, like every six months we have to go for medical check-up. (F3, translated from Malay) Sometime we are busy and forgot. (F5, translated from Malay) Like a reminder for birthday, ‘treet treet, today is your birthday’.”

(F6, translated from Malay)

“I input my health profile and the app detected that my blood pressure is slightly high. Then, there should be a reminder for me, telling me that you know, let's concentrate on reducing the salty things for today, or sugar or reduce smoking if I'm a smoker, don't take curry, don't take coconut milk, reduce your sweetened drinks or whatever for this week… especially when we work we just forget about these.”

(F2)

“For me I love the app that can send a reminder to me like I’m using right now. They count your daily steps and they will send you how much calories you burn every day. I think this is quite interesting.”

(F9)

“I think a reminder will be useful because when we work, we don't think of our health, we don't think of drinking water, a simple thing that is so important. So it's simple you know, it just reminds, every one hour or two hours, it just reminds us to drink water, so just go to the pantry and all that.”

(F15)

Some suggested that the app should incorporate a health monitoring function and able to store their health data. The participants also suggested ways to improve sustained use of the app, including providing daily short health messages, giving incentives or reward and ability to function offline.

“I think another thing you can do is to store your medical information in the app. So in the apps, when you go inside you can see, ‘Ok, my sugar level that time was this, so now is this and this. You can monitor you know. So that alone encourages them to go for more screening test, isn't it?”

(I3)

“So it's like easy, in the train you can just go through short write-ups about health. If you put it in a long paragraph, they won't read it. It's like short, short messages about health and yourself, like about how to take care of your eyes; every morning drink a glass of water; short messages that benefit health.”

(I1)

“Maybe you can organize a contest so that they get something, you give them reward if they answered correctly. Maybe you can arrange the questions regarding health. Make it very interesting, like a game.”

(I5)

“If it is offline, offline installation, don’t need Wifi, is also fine. Because sometime we don’t have Wifi or ran out of internet data.”

(I4)

#### Social connectivity

Social connectivity could be another important component of the app. The participants suggested to incorporate a forum or blog into the app and it should also be able to connect to social media. This would help them to share experience, resources and motivate each other to go for health screening.

“So it would be like a forum or something? so that you can just post a question and share with peers.”

(F15)

“I think sometimes one of the good ways to expose people is to understand other people’s story. Because a lot of the blogs I’ve seen, they describe people’s past experience you know they have this pain and what happened, the reasons and sometimes there are similarities in their story and my story.”

(F8)

### Theme 3: Dissemination

#### Advertisement

To increase the uptake of the app, the participants suggested to advertise it in various locations like hospital, gym, shopping complex, café, men’s magazine and newspapers. The app can also be spread online especially via social media and messaging app such as WhatsApp. The advertisement must have attractive design and create the need for men to use the app.

“You can just send to one guy to be sent to another guy, it’s a chain reaction, you see. Facebook is a good medium nowadays; a lot of people find something from Facebook.”

(I7)

“The fastest way is through social media like Facebook. You can also have some simple links you can pass through WhatsApp.”

(I3)

#### Recommendation

Recommendations by the doctors and promotion via celebrities are one of the ways to make men use the app. The participants also suggested that the app can be promoted via health events and health groups. A good review from third parties and encouragement of usage from peers are also good strategies to promote the app.

“My dad [a doctor] shared information on which website to go to with his patients and many of them really went to have a look at it. So in my honest opinion, I think the best way is through the doctor. I think that is a strong influence.”

(F8)

“Normally I look at the reviews first, whether it’s useful and whether it suits me. If let’s say they say it is useful then only will I download it.”

(F9)

#### Incentive

Providing incentives for people who used the app is another method suggested by the participants. Reward like discount voucher, free health screening or even monetary reward might improve the dissemination and usage of the app.

“If you want people to really blast it to more people, you got to reward them. Like I will get a small bonus, commission, points or something if I spread to my friends. That bonus I can translate to a free medical check-up or something like that. The more people you recommend, the more rewards you get out of it, you know.”

(I3)

#### Integration

Rather than making men download the app, the participants suggested that it could be pre-installed and integrated into a new smart phone basic apps package The health screening app can also be integrated into existing successful apps that have huge user base. Some participants suggested that the company should integrate the app into the company healthcare policy to encourage all staff to download and use the app.

“Maybe you may install freely in the phone. Maybe you got co-link with Apple or Samsung that when people buy the package, the app is already installed. And then they must teach the customer how to run and what are benefit of this app.”

(I8)

“If you don’t key in, you won’t get your salary. It is made compulsory. Or maybe as part of Key Performance Index.”

(F16, translated from Mandarin)

## Discussion

This study identified important characteristics that men want from a health screening mobile app and they are categorized into three key themes: content, feature and dissemination. In this study, men wanted the app to contain information about health screening and health assessment functions. The information and assessment must be personalized and trustable. The app must have user-friendly features in terms of information delivery, data entry burden, sustainability and social connectivity. Regarding dissemination, men proposed that advertisements, recommendations by health professionals, and providing incentive or delivering as part of a package may help to disseminate the app.

Lack of knowledge is often reported as a barrier to health screening [5, 37–39]. Men may not understand the benefits of screening and therefore do not perceive health screening as important. It is, thus, crucial to include information about health and health screening in health screening apps. In addition, men like mobile apps because they are able to assess their health at their own convenience rather than going to a health screening center. This might address the issues of accessibility, which is an important barrier to screening as highlighted in many studies [40, 41]. This study also found that men wanted privacy when screening. This finding concurred with studies which found that men preferred home-based to clinic-based fecal occult blood testing [5, 42]. However, currently, there are no accurate tools on mobile apps to screen for blood pressure and cholesterol. Nevertheless, questionnaire-based screening for mental health conditions such as depression are available in mobile apps [43, 44]. With advancement in biosensor research, future screening apps may be able to incorporate routine screening such as blood pressure and cholesterol measurements. This will likely to improve the uptake of screening using mobile apps.

In this study, men also wanted the health screening app to assess their individual health risk. It must also be evidence-based and come from credible source. Currently, there are several evidence-based risk assessment tools, such as the Framingham Risk Score which can be used to predict individual risk of developing coronary heart disease [45]. Credible organizations such as the United States Preventive Services Task Force (USPSTF) provides evidence-based recommendations on which health screening test should be performed; these can be incorporated into the app. Currently, the USPSTF recommends that men should be screened for hypertension, diabetes, dyslipidemia, colorectal cancer, HIV, sexually transmitted infections, hepatitis, depression, smoking, alcohol and obesity for men [46]. These, however, need to be tailored according to the individual’s age, ethnicity, past personal medical history, family history and lifestyle to avoid medical overuse [47–49].

In addition, usability of mobile health apps affects users’ decision to use the app. Nielsen states that a product with good usability must be easy to learn, efficient to use, easy to remember, have few errors and subjectively pleasing [50]. A health screening mobile app often contains medical information that may be difficult for users to understand. Therefore, it is important to consider carefully how the information will be delivered when developing the app. Another important barrier to using and sustaining a mobile health app is data entry burden, which was found to be the main factor for deleting a downloaded app [31]. Thus, when designing a health screening app, the developer should strike a balance between information accuracy and data burden, and only include essential information in the app.

Sustainability is another important factor raised by the participants. Although regularity is an important component of health screening, screening interval of some of the health conditions, such as colorectal cancer, can be long up to once every five years [46, 51]. Therefore, men may not be accessing the app regularly and this increases the chance of the app being deleted. This is compounded by the fact men may not be aware of the regularity concept of health screening. Men tend to procrastinate, forget or ignore subsequent health screenings [52–54]. It is, hence, important to incorporate additional features in the app, such as reminders and alerts, health monitoring, daily brief health messages and rewards to attract men’s attention so that they would continue using the app regularly.

Men also wanted social connectivity function in the health screening app. Social networks, specifically family and friends, were found to have a strong influence on men’s decision to go for health screening [55, 56]. Through social networking, men are able to share resources, experience and motivate each other to go for screening. This finding concurs with those of weight control and HIV testing apps, where users desire social networking as part of the app [57, 58]. A randomized controlled trial using social media as an intervention has been found to be effective in increasing HIV testing among men who have sex with men in Peru [21]. This reaffirms the increasing importance of including social media as a feature when developing mobile health apps.

Dissemination is often not considered in the development of health interventions such as a mobile health application [59]. The impact of a health intervention does not just depend on its effectiveness but the extent of its reach [60]. Therefore, a useful and well-designed app will remain unused if there is a lack of effective dissemination strategy. This is particularly relevant to health screening apps, where, unlike mobile health apps for fitness and diet, men often do not seek mobile apps on health screening [31]. This is partly due to low awareness of health screening. In this study, men proposed several useful ways to disseminate the app, including advertisement, recommendation by healthcare professionals, providing incentives and integration of the app. These suggestions are not unique to health screening and can be applied to most health-related mobile app. These proposed strategies are crucial to reach out to targeted populations to ensure maximal benefits gained from the app.

This study has several strengths and limitations. We interviewed men in a banking institution consisting of a broad range of socio-demography using purposive sampling. Most of the studies on mobile app development are based on experts’ opinions. This study explored the potential users’ experience at the pre-development phase. We also incorporated the dissemination concept in this study which is lacking in the current literature on mobile health. However, most of the participants were from a higher level of education and resided in an urban setting. Therefore, the findings may not be transferable to the other populations in Malaysia. Future studies should explore the opinions of experts from various backgrounds needs to be incorporated when developing the app to ensure high acceptability and effectiveness [61].

## Conclusions

This is one of the few studies that explored users’ need before a mobile app is developed. We found that men wanted the app to contain personalized and credible information to guide them in making decision about health screening. They preferred a mobile app to conventional screening services because of its convenience and privacy. They also offered insights into ways to ensure sustainability, increase social connectivity and enhance dissemination of the mobile app. Future studies on mobile app development should elicit users’ preference and need in terms of its content, features and dissemination strategies. We believe this will help to improve acceptability and increase the chance of successful implementation of a mobile app.
